# Phylogenomic and functional characterization of an evolutionary conserved cytochrome P450-based insecticide detoxification mechanism in bees

**DOI:** 10.1073/pnas.2205850119

**Published:** 2022-06-21

**Authors:** Julian Haas, Angela Hayward, Benjamin Buer, Frank Maiwald, Birgit Nebelsiek, Johannes Glaubitz, Chris Bass, Ralf Nauen

**Affiliations:** ^a^Institute of Crop Science and Resource Conservation, Department of Molecular Phytomedicine, University of Bonn, D-53115 Bonn, Germany;; ^b^Crop Science Division, Research and Development, Bayer AG, D-40789 Monheim, Germany;; ^c^College of Life and Environmental Sciences, Biosciences, University of Exeter, Penryn, TR10 9FE, United Kingdom

**Keywords:** P450, bee, neonicotinoid

## Abstract

The regulatory process for assessing the risks of pesticides to bees relies heavily on the use of the honeybee, *Apis mellifera*, as a model for other bee species. However, the validity of using *A. mellifera* as a surrogate for other *Apis* and non-*Apis* bees in pesticide risk assessment has been questioned. Related to this line of research, recent work on *A. mellifera* has shown that specific P450 enzymes belonging to the CYP9Q subfamily act as critically important determinants of insecticide sensitivity in this species by efficiently detoxifying certain insecticide chemotypes. However, the extent to which the presence of functional orthologs of these enzymes is conserved across the diversity of bees is unclear. Here we used a phylogenomic approach to identify > 100 putative CYP9Q functional orthologs across 75 bee species encompassing all major bee families. Functional analysis of 26 P450s from 20 representative bee species revealed that P450-mediated detoxification of certain systemic insecticides, including the neonicotinoid thiacloprid and the butenolide flupyradifurone, is conserved across all major bee pollinator families. However, our analyses also reveal that *CYP9Q*-related genes are not universal to all bee species, with some Megachilidae species lacking such genes. Thus, our results reveal an evolutionary conserved capacity to metabolize certain insecticides across all major bee families while identifying a small number of bee species where this function may have been lost. Furthermore, they illustrate the potential of a toxicogenomic approach to inform pesticide risk assessment for nonmanaged bee species by predicting the capability of bee pollinator species to break down synthetic insecticides.

## Significance

Bee pollinator pesticide risk assessment is a regulatory requirement for pesticide registration and is largely based on experimental data collected for surrogate species such as the western honeybee. Recently, CYP9Q3, a honeybee cytochrome P450 enzyme, has been shown to efficiently detoxify certain insecticides such as the butenolide flupyradifurone and the neonicotinoid thiacloprid. Here we analyzed genomic data for 75 bee species and demonstrated by the recombinant expression of 26 CYP9Q3 putative functional orthologs that this detoxification principle is an evolutionary conserved mechanism across bee families. Our toxicogenomics approach has the potential to inform pesticide risk assessment for nonmanaged bee species that are not accessible for acute toxicity testing.

Pollination is essential for most flowering plants and is functionally integral to the stability of ecosystems, including agroecosystems (1). While the staple crops responsible for the majority of human calorie intake are wind-pollinated, an estimated 75% of globally produced crops benefit from animal pollination (2), especially crops providing important micronutrients (3). Pollination is an ecosystem service carried out by a diverse range of animals, but insects, particularly bees, are widely recognized as the most important taxa and, as such, are considered vital to the maintenance of high agricultural productivity (2, 4). Bee pollinators include a relatively small number of species that may be managed by humans (managed bees) including honeybees, bumblebees and a handful of solitary bee species, and a large number of species that are not managed by humans (nonmanaged bees).

Bee species are not exempt from the declines in insect diversity and abundance reported in many regions of the world over the last decades (5–8). There are many drivers and stressors behind these losses, which have been described as “death by a thousand cuts” (9). One such driver is agricultural intensification, with the associated use of pesticides, especially insecticides, although the relative importance of individual stressors is still under debate (9, 10). Pesticide regulation includes a thorough risk assessment for bees, which, due to its economic importance, worldwide abundance, and accessibility, is largely reliant on the use of the domesticated honeybee, *Apis mellifera*, as a surrogate for other *Apis* and non-*Apis* bee species. Although bees are monophyletic, they are a highly diverse clade of insects comprising more than 20,000 known species, with broad differences in ecology and life history traits; as such, the appropriateness of using the honeybee as a surrogate species is a matter of intense debate (11). A recent publication applying a trait-based vulnerability analysis, across 10 bee species, concluded that based on a lower reproductive potential and higher likelihood of exposure, certain solitary bees may be more at risk from pesticides than the honeybee (12). Apart from exposure, the intrinsic toxicity of pesticides is another important determinant for their safe use. Assessment of the toxicity of insecticides used for sustainable pest control against several nontarget arthropods is conducted as part of existing regulatory requirements. This aims to identify any side effects of insecticides on beneficial insects, such as bees and, where possible, minimize their impact by ensuring appropriate label recommendations (e.g., application timing). Adverse intrinsic effects of pesticides on honeybees are well studied and under constant review and, where necessary, appropriate measures to alleviate risks are taken to avoid adverse effects to bee pollinators while facilitating sustainable pest control for growers (13–15).

Systemic insecticides acting on nicotinic acetylcholine receptors (nAChRs), such as neonicotinoids, are widely used to control highly destructive agricultural and horticultural pests (16, 17). However, concerns have been expressed about their environmental and ecotoxicological risks, including a potential role in bee pollinator declines (15, 18). In 2013, the European Commission (EC) first suspended the use of clothianidin, thiamethoxam, and imidacloprid (IMD) in bee-attractive crops (19). Subsequently, in 2018, the EC prohibited all outdoor uses due to the high level of risk to bee pollinators and amended the conditions of approval to restrict uses to only crops grown within permanent greenhouses. However, not all systemic insecticides binding to the orthosteric site of insect nAChRs are equally toxic to honeybees, with some assessed as practically nontoxic, according to standard regulatory measures such as acute oral and contact toxicity bioassays (20). For example, based on their low acute toxicity to honeybees, the *N*-cyanoamidine neonicotinoids (21, 22) and the butenolide flupyradifurone (FPF) (23) are considered “bee safe.” Surprisingly, these insecticides bind to the nAChRs of pest insects and honeybees with similar nanomolar affinity. However, they are orders of magnitude more toxic in vivo to pest insects (24, 25). Recent toxicogenomic studies of managed bee pollinators have shed light on this paradox by demonstrating that cytochrome P450 enzymes from the CYP9 family act as key molecular determinants of insecticide selectivity in these bee species by providing protection to certain insecticides from multiple different classes, including *N*-cyanoamidine neonicotinoids (24–29). More specifically, in the honeybee (24) and bumblebee, *Bombus terrestris* (29), P450 enzymes from the CYP9Q subfamily have been shown to efficiently metabolize *N*-cyanoamidine neonicotinoids but not *N*-nitroguanidine compounds, explaining the profound differences in toxicity of the two neonicotinoid chemotypes to these bee species (24–26). Meanwhile, in the red mason bee, *Osmia bicornis*, alternative but closely related P450 enzymes belonging to the CYP9BU subfamily perform a similar function (27). The importance of the CYP9Q/CYP9BU (CYP9Q-related) P450 subfamily in the detoxification of certain insecticidal chemotypes has been recently demonstrated by studies using the alfalfa leafcutting bee, *Megachile rotundata*, which lacks functional orthologs of such genes. This species was found to be up to 2,500-fold more sensitive to *N*-cyanoamidine neonicotinoids than honeybees in acute contact toxicity bioassays (30). Toxicogenomic investigations revealed that the increased sensitivity is correlated with the absence of *CYP9Q*-related genes in the genome of this species, resulting in a lack of detoxification capacity (30).

The recent findings on the role of P450s in defining the sensitivity of managed bee pollinators to insecticides lead to a number of important questions on the potential importance of these enzymes across the wide diversity of bee species. These include the following: 1) What is the level of evolutionary conservation of this important P450 subfamily in bees? 2) Do CYP9Q-related P450s, from a broad range of bee species, have the conserved capacity to detoxify certain insecticides? 3) By providing insight into key molecular determinants of bee sensitivity to insecticides, can a toxicogenomics approach be leveraged to inform pesticide risk assessment for nonmanaged and solitary bee species? Given the importance of these questions for a more holistic approach to bee pollinator pesticide risk assessment, we recruited the entirety of available public genomic and transcriptomic resources on bees (as of 2021)—covering 75 bee species from all the major bee families—to assemble and/or curate the respective CYP9 family P450 gene inventory. Phylogenetic analyses were recruited to identify potential *CYP9Q-*related P450s genes, and 26 of these from 20 representative bee species were selected for recombinant expression and subsequent biochemical characterization of their capacity to metabolize six coumarin model substrates, two neonicotinoids, and the butenolide FPF.

## Results

### Phylogenetic analyses of CYP3 clan P450s reveals a distinct branch of *CYP9Q-*related sequences across bee families.

The public databases for genomic and transcriptomic information were interrogated for data on bee species (*Anthophila*) (*SI Appendix*, Table S1). We retrieved assemblies from 75 species covering all bee families except Stenotritidae, for which no sequence information was available. Stenotritidae is the smallest bee family comprising approximately 20 species, in two genera, all of which are restricted to Australia (31). The other bee families were represented by 12 Megachilidae, 6 Andrenidae, 3 Colletidae, 10 Halictidae, 3 Melittidae, and 41 Apidae species. There is a bias in the available sequence information toward the Apidae, which is by far the largest and most well-studied of the bee families and includes honeybees (*Apis* spp.) and bumblebees (*Bombus* spp.).

**Fig. 1.**
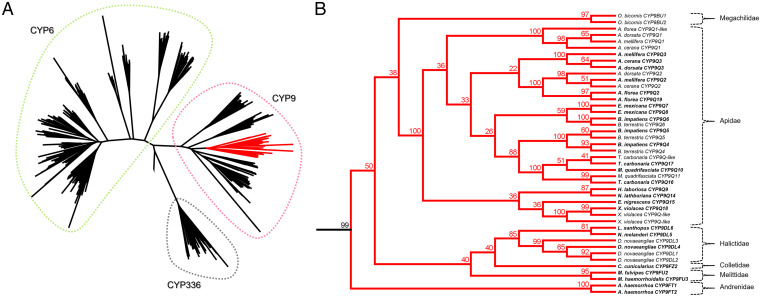
Phylogenetic relationship between CYP3 clan P450 genes from 24 bee species including all major bee families. (*A*) Phylogenetic tree of bee CYP3 clan P450 genes separated into three families: CYP6 (green), CYP336 (gray), and CYP9 (red). Apidae subfamily CYP9Q P450s including *A. mellifera* CYP9Q1 to CYP9Q3 are highlighted in red. (*B*) Detailed view of the CYP9Q-related branch with members of each of the six major bee families present. Node numbers represent bootstrap support values (%; 100 replicates). Candidate P450s selected for functional expression and characterization are highlighted in bold.

**Fig. 2.**
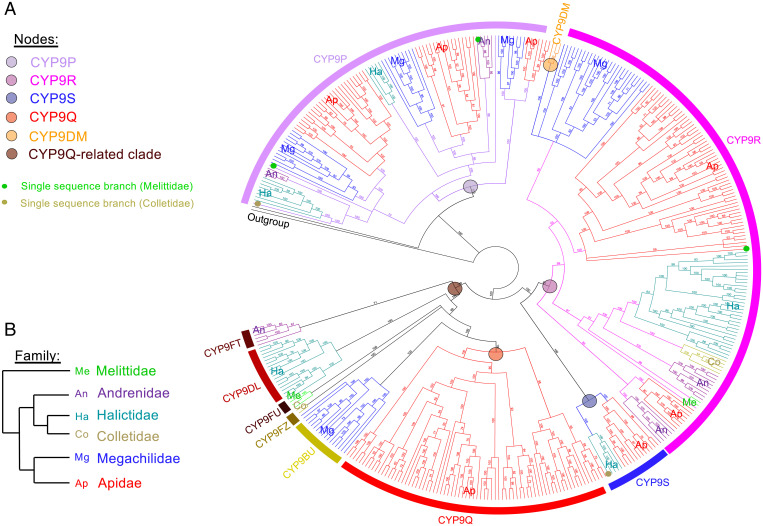
Phylogeny of the CYP9 subfamily of P450s across 75 bee species. (*A*) Phylogenetic tree of CYP9 P450s estimated using Bayesian inference. Posterior probability of nodes is shown as a % probability. Tree is rooted on *N. vitripennis* CYP9AG4 (NCBI reference sequence: NP_001166010.1). CYP9 P450s separate into distinct lineages: CYP9P (purple), CYP9S (blue), CYP9R (pink), and CYP9Q (red). CYP9Q-related proteins are further clustered into CYP9BU-, CYP9DL-, CYP9FZ-, CYP9FU-, and CYP9FT-like sequences. The small CYP9DM lineage specific to *Megachile* species is shown in orange. Sequences are colored by bee family and annotated with an abbreviated form of the family name. Single-sequence branches are labeled with a circle, colored by family. All nucleotide sequences are accessed from NCBI databases. A table with the full set of analyzed species is found in *SI Appendix*, Table S1. (*B*) Schematic of the phylogenetic relationship between bee families.

To establish the broader gene repertoire of the CYP3 clan of P450s across bee families, we selected a subset of 24 representative species with sufficient genomic information to use in phylogenetic analyses (*SI Appendix*, Table S2). In all, 579 CYP3 clan P450 sequences were identified with the resulting maximum likelihood tree revealing three distinct gene families: CYP336, CYP6, and CYP9 (Fig. 1*A*). The CYP9 family separates into five major subfamilies comprising *CYP9DN*, *CYP9R*, *CYP9S*, *CYP9P*, and *CYP9Q*-related genes. The sequences that form the *CYP9Q-*related clade include genes from all six bee families, with each family possessing a different lineage: *CYP9BU* for Megachilidae, *CYP9Q* for Apidae, *CYP9DL* for Halictidae, *CYP9FZ* for Colletidae, *CYP9FU* for Melittidae, and *CYP9FT* for Andrenidae (Fig. 1*B*). With the exception of *CYP9DN1*, which has two exons, CYP9 genes are intron-less. *CYP9DN1* is not ubiquitous across bees and is only found in 10 of the Apidae species. Where present, it is found on a different scaffold to the other four subfamilies of *CYP9*s, which are organized in a cluster at one genomic locus. To examine the organization of the four main *CYP9* subfamilies and, in particular, the distribution of *CYP9Q*-related genes in more depth, phylogenetic analyses of the sequences from the primary *CYP9* locus of 75 bee species were performed using Bayesian inference (Fig. 2). The majority of the nodes in the topology had strong posterior probability support (> 80%), and despite the fact that the support values dropped for some of the deeper nodes in the tree, none were lower than 52%. Within Megachilidae, *CYP9BU*-related genes do not appear to be universal, with six of the 12 species without a sequence that clustered as *CYP9BU*-related, suggesting the loss of this subfamily in some Megachilidae species (*SI Appendix*, Table S1). With the exception of five of the remaining 63 species, *CYP9Q-*related genes are ubiquitous across the other bee families. However, in this case the five species “missing” full-length *CYP9Q-*related genes had partial sequences, suggesting that their absence is a result of incomplete assemblies of transcriptomic data.

### Analysis of gene synteny reveals conserved genomic architecture of the *CYP9* locus across bee families.

**Fig. 3.**
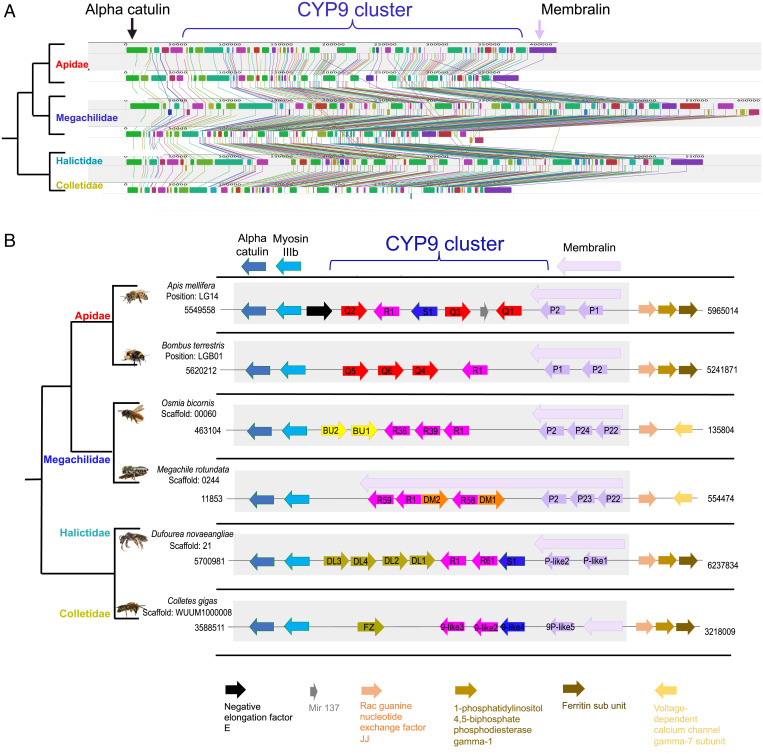
Syntenic relationship of the CYP9 loci in six bee species from four bee families. (*A*) LCBs identified at the CYP9 loci among six bee species across four families. Each colored shape is a region without rearrangement of homologous backbone sequence (a collinear block). Lines between sequences trace orthologous LCBs through the genomes. (*B*) Schematic representation of the syntenic relationship at the CYP9 loci in six bee species across four families. Arrows represent syntenic genes. CYP9 genes are colored by lineage, and the arrows denote reading frame (not drawn to scale).

The extent to which gene order and content are conserved between species (microsynteny) can provide a useful complement to sequence-based phylogenetic trees in inferring the shared ancestry of groups of genes. Six species, with good-quality genomic assemblies, were selected as exemplars of four bee families (Apidae, Megachilidae, Halictidae, and Colletidae). Scaffolds from each assembly containing the *CYP9* locus were investigated for evidence of microsynteny (Fig. 3). *CYP9Q*-related genes were found to be highly uniform in regard to their genomic position and orientation and were found in a cluster with *CYP9R* and *CYP9P* genes as part of the larger *CYP9* locus. The *CYP9* locus is framed by the same genes in all species, with *membralin* on the one side, in association with *CYP9P* genes, and *myosin IIIb* and *alpha-catulin* on the other, in association with *CYP9Q*-related genes. *Membralin* encodes an evolutionary conserved protein expressed in the central nervous system that has been reported to play a role in the endoplasmic reticulum-associated protein degradation (ERAD) pathway (32). *Myosin IIIb* encodes an actin-dependent motor protein with a protein kinase activity that in *Drosophila* is thought to play a critical role in phototransduction (33). *Alpha-catulin* encodes a cytoskeletal linker protein that facilitates Rho signaling, a pathway involved in the regulation of numerous cell processes including cell proliferation, morphology, survival, and adhesion (34).

With the exception of *CYP9Q1* in the honeybee, *CYP9Q*-related genes showed conserved gene orientation across the bee families. *Colletes gigas* was found to have the fewest CYP9 genes (five in total with only a single *CYP9Q*-related gene), and *Dufourea novaeangliae* was found to have the most (nine in total with four *CYP9Q*-related genes). It seems likely that the *CYP9* cluster emerged through tandem duplication and inversion events of an ancestral *CYP9* sequence, with additional duplication events and divergence of sequences occurring following the separation of the bee families. In *M. rotundata*, *CYP9DM*s are substituents for *CYP9BU*s in terms of genomic position and transcriptional direction. However, from a phylogenetic perspective the *CYP9DMs* appear distant to the *CYP9Q*-related sequences, the topology of the tree placing them as a sister group to the *CYP9R* subfamily (Fig. 2).

### Functional expression of CYP9Q-type P450s from different bee species across families reveals a similar metabolic profile for coumarin substrates.

To gain insight into the substrate profile of CYP9Q-type P450s identified by phylogenetic and syntenic analysis, we selected a representative panel of 26 P450 genes from 20 different bee species for heterologous expression in vitro and examined their capacity to metabolize model coumarin substrates (*SI Appendix*, Tables S3 and S4). We included P450s of at least one species belonging to all major bee families (i.e., Apidae *n* = 16 (including all major subfamilies), Andrenidae *n* = 2, Colletidae *n* = 1, Halictidae *n* = 3, Melittidae *n* = 2) but excluded the two Megachilidae managed pollinators, *O. bicornis* and *M. rotundata*, in these analyses because P450s of these species have previously been investigated in detail for their capacity to metabolize xenobiotics, including *N*-cyano neonicotinoids and FPF (28, 30, 35). All selected candidate P450s share between 44% and 88% predicted protein sequence identity with *A. mellifera* CYP9Q3 and possess the common P450 consensus and signature motifs (*SI Appendix*, Figs. S1 and S2). They showed conservation of the helix C WxxxR, helix K Ex[LM]R consensus sequences and the heme binding domain signature motif FXXGXRXCXG, whereas minor divergence was detected for a few P450s in the consensus sequences of the helix I motif Gx[ED][TS][VI] and the PERF motif PxxFxP[ED]RF (*SI Appendix*, Fig. S2).

**Fig. 4.**
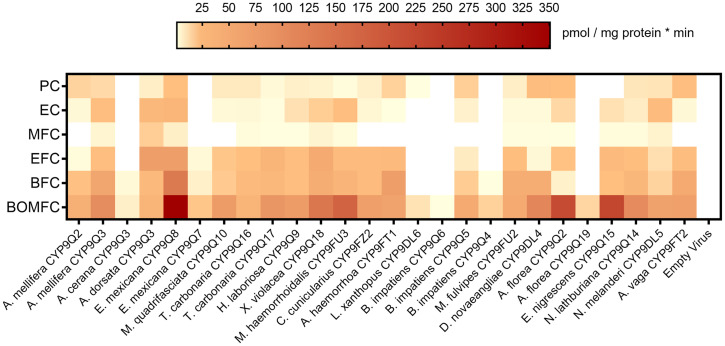
Heat map displaying the coumarin substrate profile of 26 recombinantly expressed CYP9Q-related P450s from 20 different bee species. Metabolism of selected coumarin model substrates resulting in fluorescent 7-hydroxy-4-(trifluoromethyl) coumarin (BOMFC, BFC, EFC, MFC) and 7-hydroxy coumarin (EC, PC), respectively. Data are mean values (*n* = 4). A table including all calculated values is found in *SI Appendix*, Table S5.

Using a baculovirus-mediated expression system in insect (*Trichoplusia ni*) cells, we were able to successfully express 25 of the 26 P450 enzymes and demonstrate their capacity to metabolize at least one fluorescent coumarin model substrate (Fig. 4 and *SI Appendix*, Table S5). Carbon monoxide (CO)–difference spectra exhibited a 450 nm peak for almost all the enzymes, allowing P450 quantification covering a range from 3 to 123 pmol per mg microsomal protein (*SI Appendix*, Table S6). Despite rather low expression levels of some P450s, such as *Andrena hemorrhoa* CYP9FT1 and *Apis cerana* CYP9Q3, we detected significant activity against some coumarins, so we included them in follow-up experiments with insecticides (*SI Appendix*, Table S6). Only *Bombus impatiens* CYP9Q6 could not be successfully expressed. This was substantiated by the absence of a 450 nm peak in the CO-difference spectra and a lack of activity against the entire range of coumarins tested (i.e., level of activity only marginally above that of the negative control comprising microsomal preparations isolated from cells coinfected with an empty plasmid bacmid and *A. mellifera* cytochrome P450 reductase). We therefore excluded this P450 from additional experiments (*SI Appendix*, Tables S5 and S6). However, *B. impatiens* CYP9Q5 was expressed and confirmed to be active.

In general, CYP9Q-related enzymes across bee species showed a similar preference for coumarin substrates. A stronger affinity was observed for fluorinated coumarins than for nonfluorinated analogs, and bulkier O-arylated coumarins were metabolized more effectively than O-alkylated coumarins. In keeping with earlier results for honeybee CYP9Q3, the highest enzyme activity across the CYP9Q-related P450s was detected against 7-benzyloxymethoxy-4-trifluoromethyl coumarin (BOMFC) followed by 7-benzyloxy-4-trifluoromethyl coumarin (BFC) (24). A pairwise comparison revealed that coumarin substrate profiles between most recombinantly expressed P450s were highly correlated, with the exception of *Apis dorsata* CYP9Q3, *A. hemorrhoa* CYP9FT1, and *Macropis fulvipes* CYP9FU2 (*SI Appendix*, Fig. S3).

Following the identification of model coumarin substrates for the analyzed CYP9Q-related enzymes, we employed a fluorescent probe assay that was recently described for honeybee CYP9Q2 and CYP9Q3 to screen for the interaction between BOMFC and insecticides, measuring competitive/noncompetitive insecticide-mediated inhibition of BOMFC degradation (36). We selected a single bee P450 to represent the major bee families, with the exception of Megachilidae for the reasons outlined above, i.e., *Xylocopa violacea* (Apidae) CYP9Q18, *Melitta hemorrhoidalis* (Mellitidae) CYP9FU3, *Colletes cunicularius* (Colletidae) CYP9FZ2, *D. novaeangliae* (Halictidae) CYP9DL4, and *Andrena vaga* (Andrenidae) CYP9FT2. It has recently been demonstrated that the butenolide insecticide FPF binds to honeybee CYP9Q2 and CYP9Q3 and interferes with BOMFC degradation (25). Here we found that these findings can be extended to P450s from CYP9Q-related subfamilies present in other bee families. FPF noncompetitively inhibited BOMFC metabolism catalyzed by the five tested enzymes, thus indicating enzyme–FPF interaction (*SI Appendix*, Fig. S4 and Table S7). Notably, Hanes–Woolf plots of Michaelis–Menten kinetics data of all five enzymes suggested heterotropic interaction between FPF and BOMFC (*SI Appendix*, Fig. S4), possibly indicating allosteric behavior and the presence of multiple binding sites, similar to previous findings with honeybee CYP9Qs (36).

### Phylogeny correlates with functional conservation of CYP9Q-related insecticide metabolism despite sequence diversity.

**Fig. 5.**
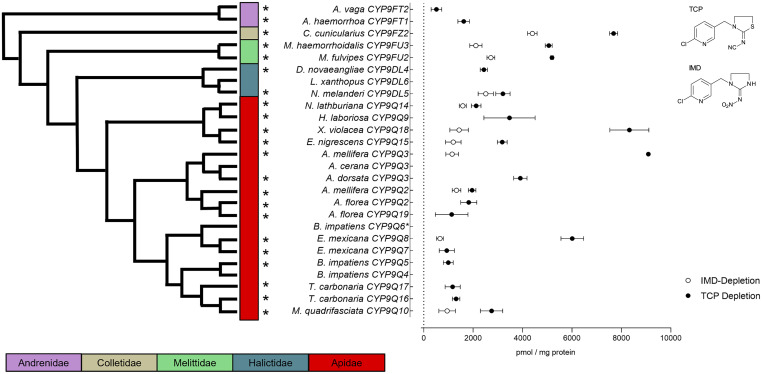
TCP and IMD depletion by 26 recombinantly expressed bee P450s. The phylogenetic relationship displayed is based on a maximum likelihood tree covering only the 26 P450s analyzed (branch length does not mirror actual distances). B. impatiens CYP9Q6* is excluded from the analysis due to failed expression. Insecticide depletion was measured after 2 h by UPLC-MS/MS and expressed in pmol/mg protein. Data are mean values ± SD (*n* = 3). Missing data points indicate nonsignificant neonicotinoid depletion compared to controls without NADPH (*P* > 0.05, unpaired *t* test). Asterisks indicate significant differences between IMD and TCP depletion for each P450 analyzed (*P* < 0.05, unpaired *t* test).

Following the indirect confirmation of an interaction between FPF and various CYP9Q-related representative P450s, we used ultra-performance liquid chromatography-tandem mass spectrometry (UPLC-MS/MS) to investigate insecticide metabolism in more detail. First, we asked whether, in common with honeybee CYP9Q3 (24), the CYP9Q-related enzymes expressed in this study can metabolize the *N*-cyanoamidine neonicotinoid thiacloprid (TCP) with high efficiency, in comparison to the *N*-nitroguanidine neonicotinoid IMD. We observed that 22 out of the 25 recombinantly expressed CYP9Q-related P450s showed significant depletion of TCP compared to controls (−NAPDH), ranging from 511 pmol per mg protein (SD ± 214; *A. vaga* CYP9FT2) to 9,092 pmol per mg protein (SD ± 66; *A. mellifera* CYP9Q3), with an average depletion across all analyzed P450s of 3,357 pmol per mg protein (Fig. 5). In contrast, only 11 out of 25 enzymes exhibited significant IMD depletion compared to controls void of the cofactor NADPH. Furthermore, for all CYP9Q-related P450s, TCP depletion was significantly greater than IMD depletion (unpaired *t* test, *P* < 0.05). Hydroxy-imidacloprid (IMD-OH) was the main metabolite identified accounting for most of the IMD depletion. Notably, for TCP some of the enzymes showed a significant gap between hydroxy-thiacloprid (TCP-OH) quantity and TCP depletion, indicating the formation of additional metabolites not covered in our analysis (*SI Appendix*, Table S8).

**Fig. 6.**
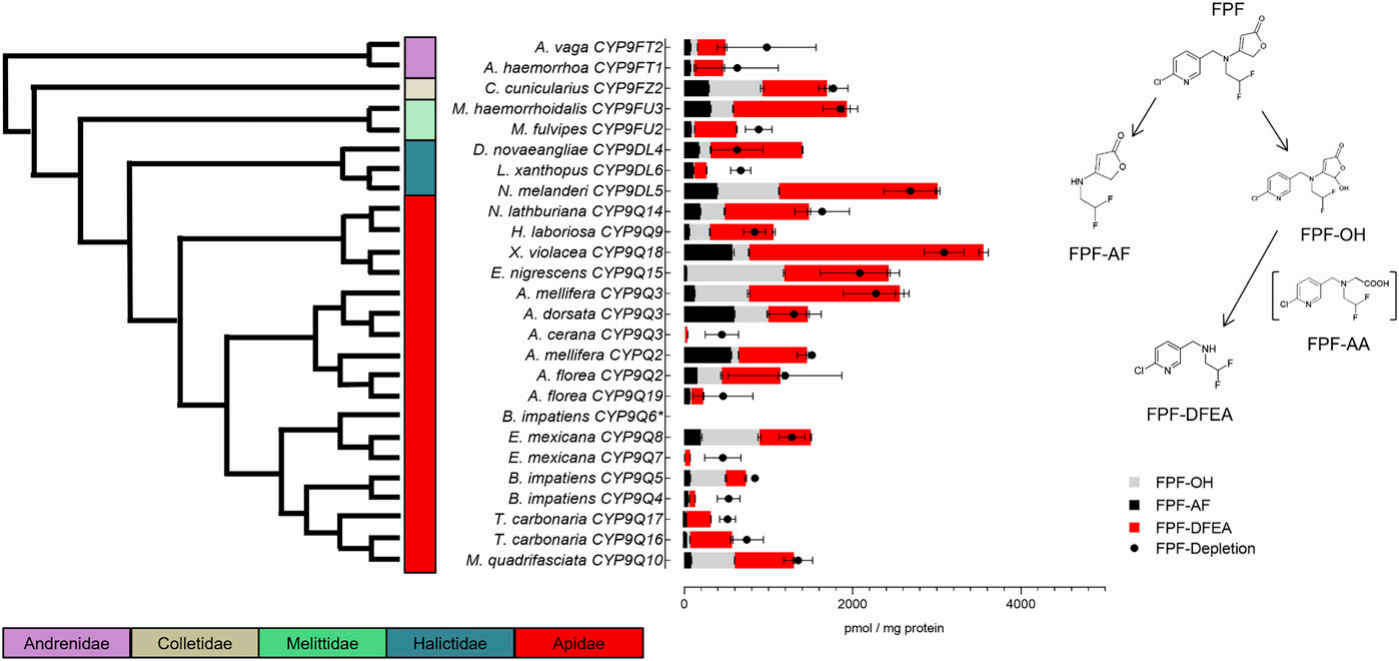
FPF metabolism and depletion by 26 recombinantly expressed bee P450s. Metabolites identified were FPF-OH, FPF-DFEA, and FPF-AF. The phylogenetic relationship displayed is based on a maximum likelihood tree covering only the protein sequences of the 26 P450s analyzed (branch length does not mirror actual distances). Insecticide depletion was measured after 2 h by UPLC-MS/MS and expressed in pmol/mg protein. Data are mean values ± SD (*n* = 3). B. impatiens CYP9Q6* is excluded from the analysis due to failed expression. The metabolic fate of FPF is based on Haas et al. 2021 (25).

As recently demonstrated, honeybee CYP9Q2 and CYP9Q3 are essential for the oxidative metabolism of FPF by two metabolic pathways leading to the metabolites FPF-4-[(2,2- difluoroethyl)amino]-furanone (FPF-AF), FPF-hydroxy (FPF-OH), and FPF-difluoroethanamine (FPF-DFEA), which are not acutely toxic (25). Here, our results demonstrated that FPF metabolite formation was remarkably uniform among all the P450s tested, suggesting conserved FPF detoxification pathways across bee species (Fig. 6). Hydroxylation and subsequent degradation of the furanone moiety was the preferred oxidative metabolic fate confirmed for all tested bee P450s, resulting in FPF-DFEA as the major metabolite followed by FPF-OH and FPF-AF. Average FPF depletion was 1,227 pmol per mg protein, ranging from 447 pmol per mg protein (SD ± 198; *A. cerana* CYP9Q3) to 3,086 pmol per mg protein (SD ± 238; *X. violacea* CYP9Q18).

## Discussion

The *CYP9Q* P450 subfamily of honeybees matches many characteristics of gene subfamilies involved in environmental response and xenobiotic detoxification. Its members are expressed in tissues and organs involved in the detoxification process (24, 37) and across life stages of bees that are exposed to xenobiotics (25, 37). Furthermore, the apparent broad substrate specificity of its members is linked to potential multiple substrate binding sites (36), which is a known feature of xenobiotic-metabolizing P450s (38, 39). Finally, CYP9Q3 has been shown to be a key molecular determinant of insecticide selectivity in *A. mellifera* and, remarkably, is capable of metabolizing different insecticidal chemotypes belonging to five different insecticide classes including pyrethroids, neonicotinoids, organophosphates, diamides, and butenolides (24–27). Beyond honeybees, related genes, such as CYP9Q4/5/6 and CYP9BU1/2 in bumblebees and red mason bees, respectively, have been shown to have a similar profile and capacity to detoxify insecticides (24, 28, 29). Taken together, these findings suggest that just as in humans, in whom the P450s CYP3A4 and CYP2D6 are together responsible for the metabolism of > 50% of clinically used drugs (40), a handful of key P450s in bees may be important metabolizers of natural and synthetic environmental xenobiotics. However, recent work has demonstrated that not all bee species have CYP9Q-related P450s, with the managed pollinator *M. rotundata* lacking such enzymes. This was found to have profound implications for the sensitivity of *M. rotundata* to insecticides, with this species displaying > 2,500-fold greater sensitivity to the neonicotinoid thiacloprid than other managed bee pollinators. Given these findings, it is imperative to understand which species of bees have P450 enzymes that provide protection against certain insecticides and which do not.

In this study we addressed this important knowledge gap. Our analysis of bee genomic resources, covering 75 species, revealed the presence of *CYP9Q* functional orthologs in species across all the major bee families. However, our data also provide further evidence that *CYP9Q*-related genes are not universal to all bee species, with six out of the 12 species within Megachilidae lacking a sequence that clustered as *CYP9Q*-related, suggesting the loss of this subfamily in certain Megachilidae species. Previous work has demonstrated that in the case of the Megachilidae species *O. bicornis*, P450s belonging to the CYP9BU subfamily, with recent shared ancestry to the Apidae CYP9Q subfamily, metabolize TCP in vitro and confer tolerance to this compound in vivo (28). It is therefore notable that two other *Osmia* species (*Osmia cornuta* and *Osmia lignaria*) were also found to have P450s belonging to this subfamily in the current study. However, our syntenic analyses of the *CYP9* locus across bee families highlighted that the *Megachile* species *M. rotundata* has *CYP9DM* genes rather than the insecticide-detoxifying *CYP9BU* genes found in *O. bicornis*. The increased sensitivity of *M. rotundata* to TCP and FPF suggests that *CYP9DM* genes have lost the capability to detoxify such compounds due to divergent evolution (30). This concept is supported by the fact that native microsomes (a source of total cytochrome P450 enzymes localized to the endoplasmic reticulum) of this species lack the capacity to metabolize TCP and FPF (30). Together, these findings provide an important example that the absence of *CYP9Q*-related P450s in bee species can be associated with an inability to metabolize certain insecticide chemotypes in vitro and high sensitivity to the same compounds in vivo (30). Thus, further analysis of Megachilidae species is warranted to understand the extent to which individual *Megachile* species lack *CYP9Q*/*CYP9BU*-related genes and the implications of this absence for their sensitivity to insecticides.

As previously shown for honeybees (41), *CYP9Q*-related genes are part of a bigger CYP9 cluster across bee families that seems to have arisen before the evolutionary separation of bee families. The presence of gene clusters is a common feature of P450 genes and is often observed for genes involved in environmental response and xenobiotic detoxification, such as those P450s found in the arthropod CYP3 clan (42, 43). The CYP9 cluster in bees also shows characteristics that are typical for environmental response genes: duplication events and rapid rates of evolution (42). *CYP9Q*-related genes show recent duplication events in some but not all species, with up to five genes in *Friesomelitta varia*. Rapid rates of evolution (i.e., sequence diversity) are especially high for *CYP9Q*-related genes, effectively leading to the annotation of a distinct CYP9Q-related subfamily for each bee family (< 55% sequence identity). Therefore, it is difficult to identify them as functional orthologs based on sequence identity alone. However, our phylogenetic, syntenic, and functional analyses have provided compelling evidence for an evolutionary conserved role of such genes (44). Specifically, our data revealed a conserved functional role of CYP9Q-related enzymes in insecticide metabolism across more than 20 different bee species, including important stingless Apidae species such as *Melipona quadrifasciata* (native to Brazil) and *Tetragonula carbonaria* (endemic to Australia), and important Halictidae species such as *Nomia melanderi* (an alkali bee native to the United States). In all cases, our results revealed a common capacity to degrade TCP and to sequentially metabolize FPF.

The observed preference for TCP over IMD and the conserved sequential oxidative metabolism of FPF across CYP9Q-related enzymes of all bee species investigated provides clear evidence for functional conservation in terms of insecticide detoxification capacity. Similar conservation of xenobiotic metabolism within an insect P450 subfamily has been previously reported for the CYP6AE, CYP6B, and CYP9A subfamilies in the cotton bollworm, *Helicoverpa armigera* (45, 46), while the only reported examples for insect P450 families with functionally validated detoxification capacity across related species, to the best of our knowledge, are CYP6B orthologs in Papilionidae (swallowtail butterflies) (47) and CYP9A orthologs in some noctuid pests (46, 48). However, studies in Papilionidae dealt with a rather narrow phylogenetic range investigating the differences in furanocoumarin metabolism between closely related species from the same genus (47). Here, we demonstrate a case of conserved detoxification capacity across bee families that diverged more than 100 million years ago.

CYP9Qs are among the largest subfamilies in the CYP3 clan of bees after the CYP6AS subfamily, with up to five members in bee species (49) (*SI Appendix*, Table S1). In lepidopteran species, diversification at the P450 subfamily level is an indicator of host range expansion and thus specialization (50). In bees, the expansion of the CYP6AS family has been linked to the transition from carnivory to florivory and eusocial resin-collecting behavior, but no such pattern is evident for the CYP9Q subfamily (49). This is consistent with a recent study in which no relationship between the P450 repertoire and bee ecology was identified (51). Similarly, no obvious pattern emerged between bee families, life history traits, or dietary spectrum and the capacity of CYP9Q-related enzymes to metabolize the tested compounds in the present study. CYP9Q enzymes have been shown to metabolize the flavonol quercetin at lower rates than members of the CYP6AS family (26), leading Johnson et al. to suggest that CYP9Qs might have a broader substrate profile than CYP6AS enzymes, which are optimized for flavonoid defense in honeybees (49). The results provided in this study provide additional evidence that CYP9Q-related enzymes are key components of the response against diverse xenobiotics rather than specialized enzymes that provide defense for a single ecology-related type of chemistry.

The approach used in this study demonstrates the power of phylogenomic and syntenic analysis of genomic data to identify bee P450 genes that are putative functional orthologs of known insecticide metabolizers. With the exponential increase in genomic data being generated for insects, including bees, this approach has immense potential to inform pesticide risk assessment and avoid negative bee–pesticide interactions. Specifically, we envisage these approaches having utility to 1) explore the appropriateness of surrogate bee species in current risk assessment frameworks, 2) inform decisions on which bee species should be prioritized for toxicity testing, 3) predict and avoid negative outcomes of pesticide use on bees, and 4) facilitate the rational design of future insecticides. We briefly expand on these points below.

Currently a handful of managed bee species are used as a proxy for other species in pesticide risk assessment. By significantly advancing our understanding of the extent to which these model species are accurate representatives of nonmodel species, the research generated in this study will aid in the development of robust risk assessment frameworks. Our data illustrate the promise of leveraging phylogenetic and syntenic approaches to predict acute bee toxicity to pesticides from genomic data, and we envisage that this approach could be used as a component of Tier 0 molecularly informed risk assessment. Such an approach would have parallels with molecular medicine approaches used to characterize P450–drug interactions in the pharmaceutical industry that provide important insight into organismal physiology and health (52). To employ such a phylogenetically inspired approach, some requirements must be met. First, the molecular determinants of insecticide selectivity in the surrogate species, such as CYP9Q2 and CYP9Q3 metabolizing TCP and FPF in *A. mellifera*, must be known. Available genomic data can then be screened to identify potential functionally orthologous genes in related species. In this study, we showed that none of the 41 Apidae species with sufficient genomic information available and investigated here lacks CYP9Q-related genes. This suggests, assuming functional conservation of CYP9Q-related P450s, that all these Apidae species have the potential to detoxify these compounds and may therefore show surrogate species (*A. mellifera*) sensitivity and selectivity. Subsequent functional validation of selected candidate P450s following recombinant expression would help increase the confidence in their conserved capacity to detoxify certain insecticide chemotypes, such as shown in this study for the detoxification of TCP and FPF by CYP9Q-related P450s of a diverse range of bee species belonging to the families Apidae, Halictidae, Colletidae, Andrenidae, and Mellitidae. Further investigation of the acute toxicity of these insecticides for those species with and without CYP9Q-related P450s will provide further data on the robustness of the predictions made using this approach. While a phylogenomic approach to predicting bee sensitivity to insecticides will not replace conventional toxicity trials, such an approach will significantly aid decisions on which species should be prioritized for toxicity testing while also informing pesticide risk assessment in bees not readily accessible for acute toxicity testing. Finally, as the functional characterization of bee P450s expands, the data obtained have the potential to inform tools that assess protein sequence similarity across taxonomic groups of species as a means to predict their relative intrinsic susceptibility to a chemical of interest. An example of this is the U.S. Environmental Protection Agency Sequence Alignment to Predict Across Species Susceptibility tool (SeqAPASS v6.0; https://seqapass.epa.gov/seqapass/), which extrapolates chemical toxicity knowledge across species through the evaluation of conserved protein sequence and structure (53).

A toxicogenomic approach can also be used to predict and avoid negative outcomes of pesticide use. Specifically, understanding which bees have P450s that can metabolize certain insecticides allows rational strategies to be developed for the deployment of these products that avoids or minimizes exposure to species that lack these protective enzymes. Furthermore, the panel of recombinant P450s developed in this study can be used to screen existing pesticides to identify and avoid harmful synergistic pesticide–pesticide interactions that inhibit these enzymes. The utility of this approach has been recently demonstrated using recombinant honeybee CYP9Q3 to explain the synergistic effects between insecticides and fungicides observed at the phenotypic level (36).

Finally, the tools generated here can be used in the development of next-generation bee-safe insecticides. Specifically, the recombinant P450 panels provide a filtering tool to examine the metabolic liability of future lead compounds to understand if they are likely to be rapidly metabolized by bees. This development is of high value as live bioassays on bees are expensive and time-consuming to perform, and it is only possible to screen honeybees and solitary species for a few months of the year. In contrast, recombinant P450 panels are inexpensive and rapid to use, and they can be employed year-round.

In conclusion, our results reveal an evolutionary conserved capacity of CYP9Q-related P450s to metabolize certain insecticides across all major bee families and illustrate the promise of a toxicogenomics approach in informing bee pollinator pesticide risk assessment for nonmanaged bee species.

## Materials and Methods

### Chemicals.

FPF, FPF-AF, FPF-acetic acid, FPF-DFEA, FPF-OH, TCP, TCP-OH, IMD, and IMD-OH were of analytical grade and obtained in-house (Bayer AG, Monheim, Germany). The fluorescent probe BOMFC (CAS 277309–33-8) was custom-synthesized by Enamine Ltd. (Riga, Latvia) with a purity of 95%. In addition, 7-pentoxy-coumarin (PC) was synthesized in-house (Bayer AG, Monheim, Germany). Other coumarin model substrates and products (7-ethoxy-coumarin [EC], 7-ethoxy-4-(trifluoromethyl)-coumarin [EFC], 7-methoxy-4-(trifluoromethyl)-coumarin [MFC], BFC, 7-hydroxy-coumarin [HC; CAS: 93–35-6, purity 99%], and 7-hydroxy-4-trifluoromethylcoumarin [HFC; CAS: 575–03-1, purity 98%]) were of analytical grade and purchased from Sigma-Aldrich. NADPH-reduced tetrasodium salt hydrate (CAS: 2646–71-1 anhydrous, purity ≥ 93%) was also obtained from Sigma-Aldrich. Unless otherwise mentioned, all other chemicals were of analytical grade and obtained from Sigma-Aldrich (St. Louis, MO).

### Bioinformatic and phylogenetic analysis.

Genomic and transcriptomic assemblies from bee species (Apidae [bees]; taxid: 34735) were retrieved from the National Center for Biotechnology Information (NCBI) database (https://www.ncbi.nlm.nih.gov/) (*SI Appendix*, Table S1). Cytochrome P450 sequences were identified by querying proteins for the conserved cytochrome P450 domain (Pfam: PF00067) using InterProScan (54) and Blast2GO (55). Clade 3 P450s from 24 species were selected, and protein sequences were aligned using the MUSCLE algorithm (56) in Geneious (version 10.2.6, Biomatters, New Zealand). Alignments were used to generate a maximum likelihood tree using PhyML (57) with JTT as the substitution model and 100 bootstraps.

To generate the *CYP9* phylogeny, the nucleotide sequences for *A. mellifera CYP9Q3* (XM_006562300.3), *CYP9P1* (XM_006562302.3), and *CYP9R1* (XM_026445177.1) were used as the query sequences in a BLASTN search through the assembly of the genome or transcriptome of each bee species to identify *CYP9* homologs. A TBLASTN translated protein similarity search was also performed using the same query sequences. All resulting hit tables were downloaded. Scaffolds containing *CYP9* P450s were downloaded as a GenBank (full) file and imported into Geneious. Unannotated *CYP9* sequences were found using the “find in document” tool implemented in Geneious and the BLASTN alignment results. *CYP9* sequences for each bee species were translated and inspected for the presence of conserved motifs. Partial sequences and those that contained stop codons were removed. The resulting CYP9 protein sequences were aligned with the outgroup sequence CYP9AG4 from *Nasonia vitripennis* (NP_001166010.1) in Geneious using MUSCLE (default settings). CYP9AG4 was selected as an outgroup sequence as it is from a Hymenopteran species that diverged well before bees arose, but it still showed sufficient homology to provide a reliable alignment, i.e., it exhibited > 40% identity to most other sequences in the alignment (range, 16.35 to 50.2%). MEGAX (58) was used to find the best-fit model of amino acid substitution, using a maximum likelihood fit of 56 different models. Parameters including substitution model, proportion of invariable sites, and rate variation were calculated. The substitution model with the lowest Bayesian Information Criterion score was selected for use in phylogeny estimation. The alignment was used to generate phylogeny using Bayesian inference (59) (substitution model: LG+G [60]; chain length: 1,100,000; subsampling frequency: 200; burn-in length: 100,000; heated chains: 4; heated chain temperature: 0.2).

Analysis of synteny was conducted as follows: Genomic sequences containing *CYP9* sequences were retrieved from the NCBI database (https://www.ncbi.nlm.nih.gov/) for *A. mellifera* (DH4 linkage group LG14, Amel_HAv3.1 WGS), *B. terrestris* (LG B01, Bter_1.0 WGS), *M. rotundata* (MROT_1.0: scf_0244), *O. bicornis* (Obicornis_v3: scf00060), *D. novaeangliae* (ASM127255v1: scaffold21), and *C. gigas* (ASM1312311v1: WUUM01000008). Synteny analysis between these scaffolds (macrosynteny) was performed using Mauve (multiple alignment of conserved genomic sequence with rearrangements) version 2.4.0 (61, 62). This allowed for the order and orientation of segments to be displayed and all locally collinear blocks (LCBs) to be outlined. The region ∼200 Kbp upstream and downstream of the *CYP9* genes was examined in more detail for microsynteny. The region containing the *CYP9* cluster in *A. mellifera* was used as the reference, the annotated scaffolds were examined manually, and flanking genes were noted. For a region to be considered as showing microsynteny, the minimum requirement was the conservation of two neighboring homologs with no more than five unrelated genes in the intervening DNA. The *C. gigas* genome was unannotated. To identify the flanking genes, a BLAST database was created from the scaffold (ASM1312311v1: WUUM01000008) and flanking genes from *D. novaeangliae* were used as query sequences in a discontinuous BLASTN search. The first and last exons were identified, and genes were annotated in Geneious.

### Functional expression of P450s and metabolism assays.

Functional expression of recombinant P450 proteins (*SI Appendix*, Table S4) was conducted in High-5 insect cells as previously described (24, 25). This cell line was selected because it does not exhibit background levels of neonicotinoid metabolism as observed for other cell lines (63). All P450s were coexpressed with *A. mellifera* NADPH-dependent cytochrome P450 reductase (CPR) (accession number: XP_006569769.1).

Activity of isolated membrane fractions was tested using six fluorescent coumarin model substrates. Assays were performed in flat-back, black 384-well microplates with 50 μL total reaction volume and four technical replicates. Assay conditions and fluorescence readout were as recently described (64) with slight modifications: BOMFC was tested at 50 µM final concentration, and microsomal preparations isolated from High 5 cells coinfected with an empty plasmid bacmid and *A. mellifera* CPR served as a negative control. Fluorescent probe kinetic assays were done exactly as described previously (36). Relative fluorescent units were converted into pmol HC/HFC by generating a standard curve of the fluorescent products. CO-difference spectra of recombinantly expressed proteins were determined as previously described (65) using a Specord 200 Plus Spectrophotometer (Analytik Jena, Jena, Germany).

For insecticide depletion and metabolite quantification, incubation assays and subsequent UPLC-MS/MS analysis was performed exactly as described before (24, 25). Briefly, 80 µg microsomal protein was incubated in 100 µL reaction volume with 10 µM FPF, TCP, or IMD in the presence of the NADPH regeneration system (Promega, 1.3 mM NADP+, 3.3 mM glucose-6-phosphate, 3.3 mM MgCl_2_, and 0.4 U/mL glucose-6-phosphate dehydrogenase) for 2 h at 30 °C. Controls included replicates without a regeneration system and incubation of microsomal preparations isolated from insect cells infected with an empty baculovirus. For the chromatography on an Agilent 1290 Infinity II, a Waters Acquity HSS T3 column (2.1 × 50 mm, 1.8 mm) with acetonitrile/water/1% formic acid as the eluent in gradient mode was employed. After positive electrospray ionization, ion transitions were recorded on a Sciex API6500 Triple Quad. FPF, TCP, IMD, and their metabolites were measured in positive ion mode. The peak integrals were calibrated externally against a standard calibration curve. Ion transitions and the linear range for quantification are available in *SI Appendix*, Table S9. Recovery rates of parent compound in −NADPH samples were normally close to 100%. Obtained concentrations were converted into pmol parent/metabolite per mg microsomal protein. An unpaired *t* test (*P* < 0.05) was used to determine whether parent compound concentrations in +NADPH samples were significantly different from −NADPH controls. Parent depletion was calculated by subtracting the values from +NADPH samples from the average of −NADPH replicates.

Unless otherwise stated, all data were analyzed and visualized using GraphPad Prism (version 9.1.0, GraphPad Software Inc., CA). Data from P450 saturation kinetics experiments were analyzed for competitive, noncompetitive, and mixed-type inhibition by nonlinear regression assuming Michaelis–Menten kinetics.

## Supplementary Material

Supplementary File

## Data Availability

All study data are included in the article and/or *SI Appendix*.
