# Supplementary material for: Phylogenomic and functional characterization of an evolutionary conserved cytochrome P450-based insecticide detoxification mechanism in bees
Source: Proc Natl Acad Sci U S A. 2022 Jun 21;119(26):e2205850119. doi: 10.1073/pnas.2205850119 (PMC9245717; doi:10.1073/pnas.2205850119)
Supplement: Supplementary File [file pnas.2205850119.sapp.pdf]

PNAS

Supplementary Information for:
Phylogenomic and functional characterization of an evolutionary conserved cytochrome P450-based insecticide detoxification mechanism in bees

Julian Haas, Angela Hayward, Benjamin Buer, Frank Maiwald, Birgit Nebelsiek, Johannes Glaubitz, Chris Bass, Ralf Nauen

Ralf Nauen
Email: ralf.nauen@.bayer.com
Chris Bass
Email: c.bass@exeter.ac.uk

This PDF file includes:

Figures S1 to S4
Tables S1 to S9

	CYP9Q3 Apis mellifera	CYP9Q2 Apis mellifera	A. dorsata CYP9Q3	A. cerana CYP9Q3	A. florea CYP9Q19	A. florea CYP9Q2	B. impatiens CYP9Q6	B. impatiens CYP9Q5	B. impatiens CYP9Q4	E. mexicana CYP9Q8	E. mexicana CYP9Q7	T. carbonaria CYP9Q17	T. carbonaria CYP9Q16	M. quadrifasciata CYP9Q10	N. lathburiana CYP9Q14	H. laboriosa CYP9Q9	X. violacea CYP9Q18	E. nigrescens CYP9Q15	L. xanthopus CYP9DL6	N. melanderi CYP9DL5	D. novaeangliae CYP9DL4	C. cunicularius CYP9FZ2	M. haemorrhoidalis CYP9FU2	M. fulvipes CYP9FU3	A. haemorrhoa CYP9FT1	A. vaga CYP9FT2
A. Mellifera CYP9Q3	X	58	88	88	56	55	56	57	56	56	58	56	60	57	52	52	55	51	44	46	48	50	47	48	46	45
A. Mellifera CYP9Q2	58	X	60	59	87	84	59	62	60	57	57	60	62	63	56	55	55	58	45	48	49	52	50	49	47	46
A. dorsata CYP9Q3	88	60	X	91	57	57	56	57	56	58	58	57	59	57	52	51	55	52	44	48	48	49	48	48	45	45
A. cerana CYP9Q3	88	59	91	X	56	56	56	58	57	56	57	58	61	57	51	51	54	51	45	46	47	49	48	48	45	46
A. florea CYP9Q19	56	87	57	56	X	90	58	60	59	56	55	57	57	58	54	53	55	57	46	47	50	50	50	49	46	45
A. florea CYP9Q2	55	84	57	56	90	X	57	60	59	54	53	56	57	58	54	53	54	56	47	48	51	49	49	48	44	43
B. impatiens CYP9Q6	56	59	56	56	58	57	X	62	61	60	60	59	62	61	56	55	55	54	49	51	50	50	48	50	47	47
B. impatiens CYP9Q5	57	62	57	58	60	60	62	X	92	60	59	66	69	71	59	55	58	58	50	50	52	52	50	48	48	49
B. impatiens CYP9Q4	56	60	56	57	59	59	61	92	X	60	58	65	68	68	59	54	58	57	50	50	52	52	51	49	48	49
E. mexicana CYP9Q8	56	57	58	56	56	54	60	60	60	X	78	58	62	62	56	54	56	54	45	51	49	52	49	48	47	47
E. mexicana CYP9Q7	58	57	58	57	55	53	60	59	58	78	X	56	58	57	55	54	56	54	46	49	49	51	51	50	48	47
T. carbonaria CYP9Q17	56	60	57	58	57	56	59	66	65	58	56	X	78	76	55	52	54	53	47	46	49	49	46	46	45	46
T. carbonaria CYP9Q16	60	62	59	61	57	57	62	69	68	62	58	78	X	87	58	55	57	56	48	50	51	52	49	49	48	49
M. quadrifasciata CYP9Q10	57	63	57	57	58	58	61	71	68	62	57	76	87	X	57	55	57	56	46	48	50	51	49	47	48	48
N. lathburiana CYP9Q14	52	56	52	51	54	54	56	59	59	56	55	55	58	57	X	61	59	58	48	47	51	53	53	50	48	48
H. laboriosa CYP9Q9	52	55	51	51	53	53	55	55	54	54	54	52	55	55	61	X	56	56	48	49	50	53	52	49	47	48
X. violacea CYP9Q18	55	55	55	54	55	54	55	58	58	56	56	54	57	57	59	56	X	59	48	49	51	51	52	50	49	48
E. nigrescens CYP9Q15	51	58	52	51	57	56	54	58	57	54	54	53	56	56	58	56	59	X	48	47	50	50	53	50	49	49
L. xanthopus CYP9DL6	44	45	44	45	46	47	49	50	50	45	46	47	48	46	48	48	48	48	X	54	57	52	53	53	49	48
N. melanderi CYP9DL5	46	48	48	46	47	48	51	50	50	51	49	46	50	48	47	49	49	47	54	X	57	53	51	50	47	47
D. novaeangliae CYP9DL4	48	49	48	47	50	51	50	52	52	49	49	49	51	50	51	50	51	50	57	57	X	55	54	50	49	48
C. cunicularius CYP9FZ2	50	52	49	49	50	49	50	52	52	52	51	49	52	51	53	53	51	50	52	53	55	X	58	55	54	55
M. haemorrhoidalis CYP9FU2	47	50	48	48	50	49	48	50	51	49	51	46	49	49	53	52	52	53	53	51	54	58	X	61	54	54
M. fulvipes CYP9FU3	48	49	48	48	49	48	50	48	49	48	50	46	49	47	50	49	50	50	53	50	50	55	61	X	51	52
A. Haemorrhoa CYP9FT1	46	47	45	45	46	44	47	48	48	47	48	45	48	48	48	47	49	49	49	47	49	54	54	51	X	88
A. vaga CYP9FT2	45	46	45	46	45	43	47	49	49	47	47	46	49	48	48	48	48	49	48	47	48	55	54	52	88	X

Fig. S1. Protein sequence identity matrix (%) of the selected CYP9Q-related enzymes.

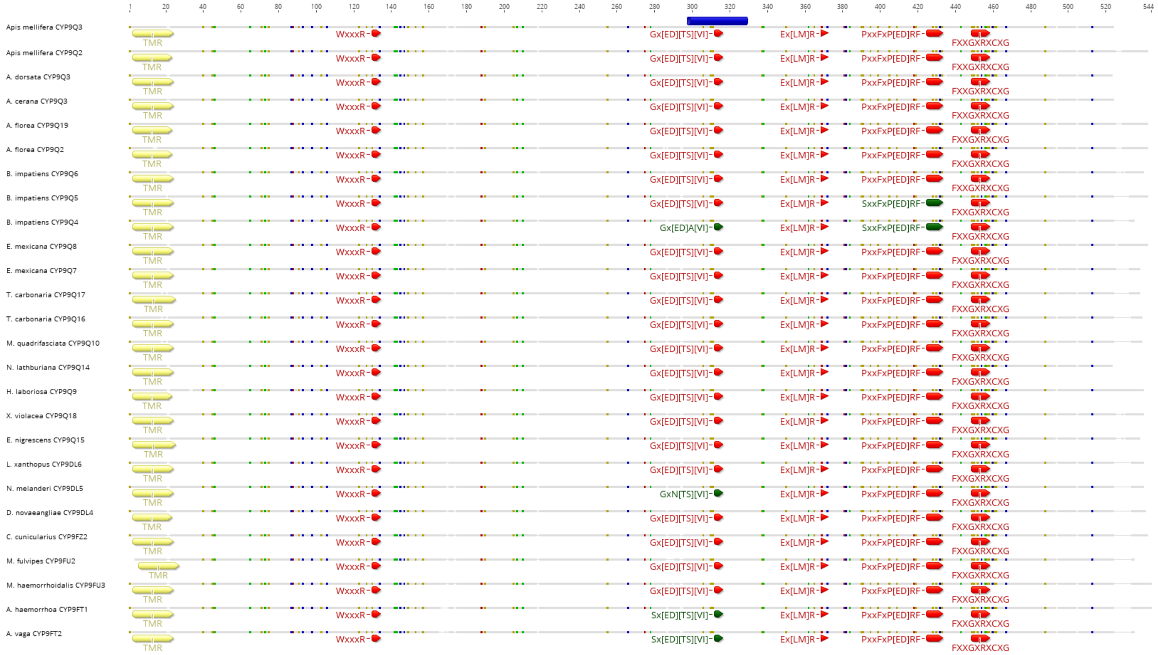

Fig. S2. Alignment of recombinantly expressed CYP9Q related enzymes with focus on the five signature P450 motifs (red) in insects with deviations in single amino acid residues highlighted in green. The transmembrane region (TMR) is depicted in yellow, and the I-helix region is marked in blue.

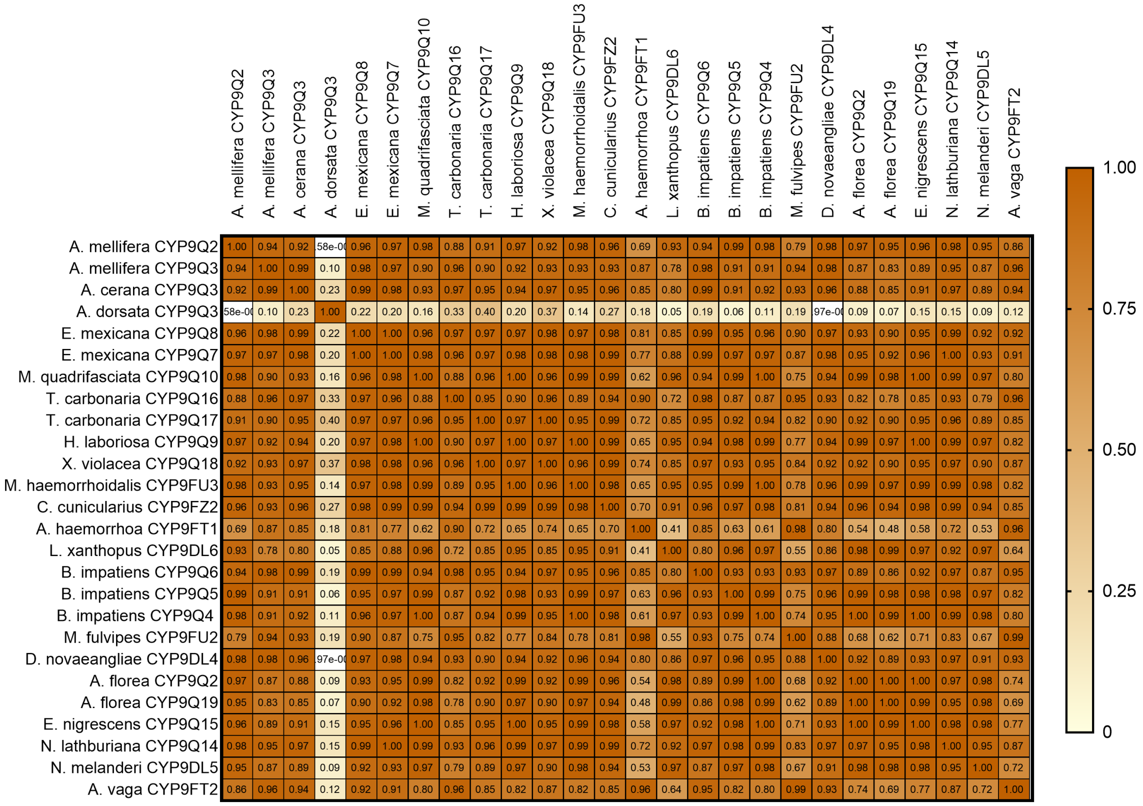

Fig. S3. Correlation matrix (pearson r) of the coumarin model substrate profle of the investigated CYP9Q-related enzymes.

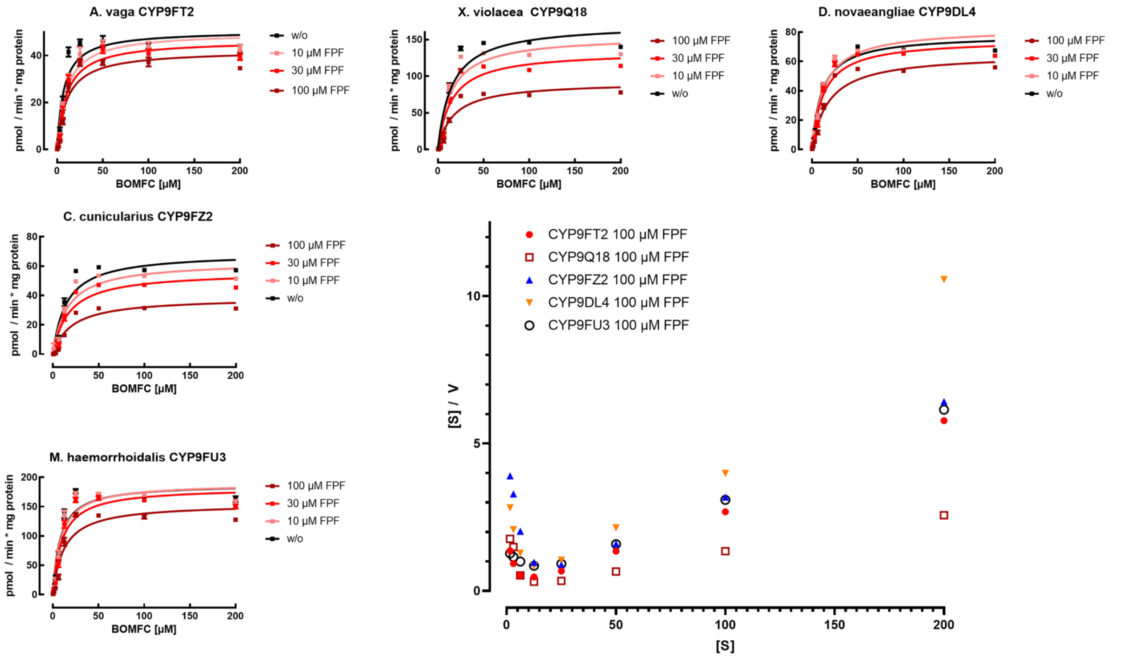

Fig. S4. Fluorescence probe assay with BOMFC and increasing concentrations of flupyradifurone (FPF). Hanes-Woolf plot (large inset) shows allosteric behavior and heterotropic interaction between FPF and BOMFC. [S] = BOMFC concentration in µM, V = velocity in pmol product / min x mg protein.

Table S1. Sequence information obtained for 75 bee species (WGS = Whole Genome Sequencing; TSA = Trancsriptome Shotgun Assembly). N/F = not found (incomplete assembly).

Species name	Family	CYP9Q-like genes	Assembly type	Assembly accession
Ammobates syriacus	Apidae	1	TSA	GBMX00000000
Andrena cineraria	Andrenidae	N/F	TSA	GHFW00000000
Andrena fulva	Andrenidae	N/F	TSA	GHFR00000000
Andrena haemorrhoa	Andrenidae	1	TSA	GHFU00000000
Andrena vaga	Andrenidae	1	TSA	GBLF00000000
Anthidium manicatum	Megachilidae	N/F	TSA	GBOJ00000000
Apis cerana	Apidae	3	WGS	JANR01000000
Apis dorsata	Apidae	3	WGS	AUPE01000000
Apis florea	Apidae	3	WGS	AEKZ01000000
Apis mellifera	Apidae	3	WGS	QIUM02000000
Bomubs bifarius	Apidae	3	WGS	JAAQOX01
Bombus campestris	Apidae	3	WGS	GCA_905332975.1
Bombus cryptarum	Apidae	3	TSA	GHGD01000000
Bombus impatiens	Apidae	3	WGS	AEQM02000000
Bombus pascuorum	Apidae	3	WGS	GHFY01000000
Bombus pyrosoma	Apidae	N/F	WGS	GGGP01000000
Bombus rupestris	Apidae	3	TSA	GBQF01000000
Bombus terrestris	Apidae	3	WGS	AELG01000000
Bombus vancouverensis	Apidae	3	WGS	JAAQRE01
Bombus vosnesenkii	Apidae	3	WGS	JAAQVK01
Camptopoeum sacrum	Andrenidae	3	TSA	GBOI01000000
Ceratina australensis	Apidae	2	WGS	SHCU01000000
Ceratina calcarata	Apidae	4	WGS	LSNX01000000
Ceratina chalybea	Apidae	2	TSA	GBPU01000000
Chelostoma florisomne	Megachilidae	1	TSA	GBPN01000000
Coelioxys conoidea	Megachilidae	N/F	TSA	GBPZ01000000
Colletes cunicularius	Colletidae	1	TSA	GBUJ01000000
Colletes gigas	Colletidae	1	WGS	WUUM01
Dasypoda hirtipes	Melittidae	1	TSA	GBMP01000000
Dioxys cincta	Megachilidae	1	TSA	GBPP01000000
Dufourea dentiventris	Halictidae	2	TSA	GBTY01000000
Dufourea novaeangliae	Halictidae	4	WGS	LGHO01000000
Epeolus variegatus	Apidae	2	TSA	GBPW01000000
Eucera nigrescens	Apidae	1	TSA	GBPG01000000
Eucera plumigera	Apidae	1	TSA	GBLY01000000
Eucera syriaca	Apidae	1	TSA	GBLZ01000000
Eufriesea mexicana	Apidae	2	WGS	LLKC01000000
Euglossa cordata	Apidae	1	TSA	SRX040737
Euglossa dilemma	Apidae	2	WGS	NIJG01000000
Eulaema nigrita	Apidae	N/F	TSA	SRX040736
Friesomelitta varia	Apidae	5	WGS	WNWW01
Habropoda laboriosa	Apidae	1	WGS	LHQN01000000
Halictus quadricinctus	Halictidae	2	TSA	GBQP01000000
Heriades truncorum	Megachilidae	2	TSA	GBQK01000000
Heterotrigoma itama	Apidae	1	TSA	ERX4231413
Hylaeus variegatus	Colletidae	1	TSA	GBPS01000000
Lasioglossum albipes	Halictidae	2	WGS	ANOB01000000
Lasioglossum xanthopus	Halictidae	1	TSA	GBPT01000000
Lepidotrigona ventralis	Apidae	2	WGS	NIPQ01000000

Lithurgus chrysurus	Megachilidae	N/F	TSA	GBMJ01000000
Macropis fulvipes	Melittidae	1	TSA	GBNX01000000
Megachile rotundata	Megachilidae	0	WGS	AFJA01000000
Megachile willughbiella	Megachilidae	0	TSA	GBQN01000000
Megalopta genalis	Halictidae	4	WGS	GELL01000000
Melipona quadrifasciata	Apidae	2	WGS	LIRP01000000
Melitta haemorrhoidalis	Melittidae	1	TSA	GBVK01000000
Nomada lathburiana	Apidae	1	TSA	GBLA01000000
Nomia diversipes	Halictidae	1	TSA	GBWP00000000.1
Nomia melanderi	Halictidae	1	WGS	REGV01000000
Osmia bicornis	Megachilidae	2	WGS	MPJT01000000
Osmia cornuta	Megachilidae	2	TSA	GHFP01000000
Osmia lignaria	Megachilidae	2	WGS	JAAOZW01
Panurgus dentipes	Andrenidae	1	TSA	GBME01000000
Sphecodes albilabris	Halictidae	N/F	TSA	GBKZ01000000
Stelis punctulatissima	Megachilidae	N/F	TSA	GBWV01000000
Systropha curvicornis	Halictidae	1	TSA	GBWF01000000
Tetragonula carbonaria	Apidae	2	WGS	GBTL01000000
Tetragonula clypearis	Apidae	1	WGS	WIUT01
Tetragonula davenporti	Apidae	2	WGS	WIUW01
Tetragonula hockingsi	Apidae	3	WGS	WIUV01
Tetragonula mellipes	Apidae	4	WGS	WINE01
Tetralonia malvae	Apidae	1	TSA	GBNI00000000.1
Tetraloniella nigriceps	Apidaea	1	TSA	SRX642804
Thyreus orbatus	Apidae	1	TSA	GBLR01000000
Xylocopa violacea	Apidae	1	TSA	GBUM01000000A

Table S2. Bee species included in the phylogenetic tree of CYP3 clan P450s.

Species name	Family	CYP336	CYP6	CYP9	CYP9Q-like genes	Assembly type	Assembly accession
Andrena fulva	Andrenidae	4	10	10	ND	TSA	GHFR00000000
Andrena haemorrhoa	Andrenidae	3	9	8	1	TSA	GHFU00000000
Andrena vaga	Andrenidae	3	7	5	1	TSA	GBLF00000000
Apis cerana	Apidae	1	18	7	3	WGS	JANR01000000
Apis dorsata	Apidae	1	17	6	3	WGS	AUPE01000000
Apis florea	Apidae	1	17	5	3	WGS	AEKZ01000000
Apis mellifera	Apidae	1	22	7	3	WGS	QIUM02000000
Bombus impatiens	Apidae	2	23	5	3	WGS	AEQM02000000
Bombus terrestris	Apidae	4	21	7	3	WGS	AELG01000000
Colletes cunicularius	Colletidae	4	14	7	1	TSA	GBUJ01000000
Dufourea novaeangliae	Halictidae	4	17	7	4	WGS	LGHO01000000
Eucera nigrescens	Apidae	1	6	3	1	TSA	GBPG01000000
Eufriesea mexicana	Apidae	2	19	8	2	WGS	LLKC01000000
Habropoda laboriosa	Apidae	1	14	5	1	WGS	LHQN01000000
Lasioglossum xanthopus	Halictidae	1	15	9	1	TSA	GBPT01000000
Macropis fulvipes	Melittidae	1	6	5	1	TSA	GBNX01000000
Megachile rotundata	Megachilidae	3	20	7	0	WGS	AFJA01000000
Melipona quadrifasciata	Apidae	6	24	9	2	WGS	LIRP01000000
Melitta haemorrhoidalis	Melittidae	1	8	5	1	TSA	GBVK01000000
Nomada lathburiana	Apidae	1	10	5	1	TSA	GBLA01000000
Nomia melanderi	Halictidae	1	12	12	1	WGS	REGV01000000
Osmia bicornis	Megachilidae	3	21	9	2	WGS	MPJT01000000
Tetragonula carbonaria	Apidae	1	19	8	2	WGS	GBTL01000000
Xylocopa violacea	Apidae	2	11	6	1	TSA	GBUM01000000

Table S3. Selected candidate bee species of six different families and some of their life traits.

Species	Family	Lifestyle	Diet preference	Nesting Behavior
Apis mellifera	Apidae	Eusocial	Polylectic	Cavity nesters above-ground
Apis cerana	Apidae	Eusocial	Polylectic	Cavity nesters above-ground
Apis dorsata	Apidae	Eusocial	Polylectic	Cavity nesters above-ground
Apis florea	Apidae	Eusocial	Polylectic	Open nesting above-ground
Eufriesea mexicana	Apidae	Putatively social		
Melipona quadrifasciata	Apidae	Eusocial	Polylectic	Cavity nesters above-ground
Tetragonula carbonaria	Apidae	Eusocial	Polylectic	Cavity nesters above-ground
Habropoda laboriosa	Apidae	Solitary	Oligolectic	Soil excavators
Xylocopa violacea	Apidae	Solitary	Polylectic	Wood excavators
Melitta haemorrhoidalis	Mellitidae	Solitary	Oligolectic	Soil excavators
Colletes cunicularius	Colletidae	Solitary	Oligolectic	Soil excavators
Andrena haemorrhoa	Andrenidae	Solitary	Polylectic	Soil excavators
Lasioglossum xanthopus	Halictidae	Solitary	Polylectic	Soil excavators
Bombus impatiens	Apidae	Social	Polylectic	Cavity nesters below-ground
Macropis fulvipes	Mellitidae	Solitary	Oligolectic	Soil excavators
Dufourea novaeangliae	Halictidae	Solitary	Oligolectic	Soil excavators
Eucera nigrescens	Apidae	Solitary	Oligolectic	Soil excavators
Nomada lathburiana	Apidae	Cuckoo	Polylectic (nectar)	-
Nomia melanderi	Apidae	Solitary	Polylectic	Soil excavators
Andrena vaga	Andrenidae	Solitary	Polylectic	Soil excavators

Table S4. CYP3 clan P450 genes used in this study, accession numbers and properties of the predicted proteins.

Species	Gene Name	Accession number	Nucleotide Sequence	Length (amino acids)	Molecular Weight (kDa)*	Isoelectric Point*
Apis mellifera	*CYP9Q2*	XP_392000		532	60.952	8.52
Apis mellifera	*CYP9Q3*	XP_006562363		517	58.891	8.58
Apis cerana	*CYP9Q3*	XP_016922294.2		517	59.246	8.83
Apis dorsata	*CYP9Q3*	XP_006613022.1		515	58.932	8.32
Apis florea	*CYPQ2*	XP_012347837.2		528	60.968	8.07
Apis florea	*CYP9Q19*	XP_031775226.1		531	60.772	8.21
Eufriesea mexicana	*CYP9Q8*	XP_017758640.1		531	60.989	8.17
Eufriesea mexicana	*CYP9Q7*	XP_017758639.1		521	59.989	8.23
Melipona quadrifasciata	*CYP9Q10*	KOX69484.1		520	59.464	8.83
Tetragonula carbonaria	*CYP9Q17*		GBTL01077204.1	522	59.219	8.45
Tetragonula carbonaria	*CYP9Q16*		GBTL01078920.1	519	59.509	8.43
Habropoda laboriosa	*CYP9Q9*	XP_017794730.1		529	60.432	9.32
Xylocopa violacea	*CYP9Q18*		GBUM01016761.1	526	59.996	9.16
Melitta haemorrhoidalis	*CYP9FU3*		GBVK01019397.1	524	59.638	8.44
Colletes cunicularius	*CYP9FZ2*		GBUJ01004521.1	523	59.269	8.38
Andrena haemorrhoa	*CYP9FT1*		GHGA01007590.1	522	58.986	8,45
Lasioglossum xanthopus	*CYP9DL6*		GBPT01021710.1	522	59.783	7.96
Bombus impatiens	*CYP9Q6*	XP_033174303.1		525	60.288	8.29
Bombus impatiens	*CYP9Q5*	XP_003486050.1		525	59.799	7.97
Bombus impatiens	*CYP9Q4*	XP_033174303.1		518	59.551	9.11
Macropis fulvipes	*CYP9FU2*		GBNX01015534.1	511	58.229	9.00
Dufourea novaeangliae	*CYP9DL4*	XP_015439019.1		522	59.543	8.36
Eucera nigrescens	*CYP9Q15*		GBPG01002643.1	524	60.289	9.12
Nomada lathburiana	*CYP9Q14*		GBLA01022698.1	513	58.820	8.18
Nomia melanderi	*CYP9DL5*	XP_031837097.1		522	59.818	8.21
Andrena vaga	*CYP9FT2*		GBLF01017912.1	513	58.464	9.18

Table S5. Activity of recombinantly expressed CYP9Q-like bee P450s on six different coumarin substrates. Values are expressed as pmol product / mg protein * min (n = 4).

	**BOMFC**		**BFC**		**EFC**		**MFC**		**EC**		**PC**	
P450	Mean	SD	Mean	SD	Mean	SD	Mean	SD	Mean	SD	Mean	SD
AmCYP9Q2	38.92	1.43	9.71	0.32	1.40	0.16	0.41	0.02	1.88	0.21	7.16	0.43
AmCYP9Q3	100.38	3.92	54.43	1.11	11.89	0.33	2.41	0.07	12.25	0.37	6.24	0.89
AcCYP9Q3	3.21	0.57	1.72	0.06	0.97	0.03	0.42	0.02	0.68	0.04	0.34	0.07
AdCYP9Q3	29.39	4.52	22.15	1.30	69.01	13.79	7.91	0.34	22.88	1.24	3.40	0.20
EmCYP9Q8	350.54	22.70	135.74	1.73	69.47	10.01	3.28	0.13	27.72	0.61	11.49	1.16
EmCYP9Q7	9.00	0.18	3.12	0.13	1.83	0.11	0.40	0.02	0.59	0.05	0.62	0.06
MqCYP9Q10	73.91	3.12	8.58	0.16	9.07	1.72	0.60	0.05	1.76	0.26	4.00	0.09
TcCYP9Q16	29.21	4.29	17.29	1.11	11.41	0.33	1.32	0.21	1.86	0.07	4.01	0.03
TcCYP9Q17	83.43	4.76	22.62	1.24	31.07	1.32	1.03	0.08	1.19	0.22	1.73	0.09
HlCYP9Q9	73.08	5.38	12.11	0.74	12.39	0.16	1.08	0.05	5.29	0.11	3.07	0.16
XvCYP9Q18	137.93	3.81	42.49	0.79	47.35	1.72	2.49	0.02	7.95	0.34	2.61	0.23
MhCYP9FU3	173.60	4.66	28.20	2.82	15.37	0.37	1.36	0.04	12.36	0.67	1.37	0.06
CcCYP9FZ2	66.99	1.16	15.42	1.23	16.24	0.92	0.84	0.07	2.18	0.12	3.05	0.20
AhCYP9FT1	60.52	1.72	68.55	1.93	20.05	0.71	0.87	0.08	1.10	0.04	7.32	0.30
LxCYP9DL6	4.95	0.15	-0.01	0.03	0.58	0.02	0.25	0.01	0.98	0.06	1.07	0.08
BiCYP9Q6	1.09	0.07	0.62	0.12	0.40	0.02	0.30	0.03	0.22	0.04	0.26	0.06
BiCYP9Q5	48.67	1.06	8.12	0.78	3.76	0.06	0.44	0.04	2.88	0.23	7.80	0.19
BiCYP9Q4	7.30	0.25	1.03	0.05	0.73	0.02	0.21	0.01	0.64	0.05	0.60	0.03
MfCYP9FU2	49.00	1.40	42.85	2.49	13.13	0.62	1.17	0.06	1.50	0.10	3.42	0.28
DnCYP9DL4	110.03	0.45	43.32	2.55	1.94	0.05	1.24	0.08	1.64	0.08	13.64	1.51
AfCYP9Q2	216.37	5.32	2.99	0.18	9.67	0.83	1.13	0.04	6.44	0.27	11.68	0.91
AfCYP9Q19	6.88	0.19	-0.19	0.04	0.34	0.02	0.31	0.02	0.45	0.07	0.43	0.04
EnCYP9Q15	224.51	5.31	11.64	0.42	19.89	2.19	1.29	0.04	5.19	0.17	0.46	0.04
NlCYP9Q14	103.12	3.72	25.68	0.87	13.02	0.90	1.37	0.07	3.58	0.10	4.53	0.12
NmCYP9DL5	62.01	2.09	7.24	0.18	5.52	0.55	2.63	0.06	15.37	1.84	4.82	0.10
AvCYP9FT2	63.60	1.06	48.70	1.08	14.18	1.27	0.89	0.07	1.86	0.11	11.86	0.75
Empty Virus	0.83	0.04	0.05	0.06	0.15	0.03	0.26	0.07	0.06	0.03	0.21	0.01

Table S6. P450 content in pmol/mg protein determined by CO-difference spectra. Abbreviation: n.d., not detected.

Species	Gene Name	pmol per mg protein
Apis mellifera	*CYP9Q2*	115.30
Apis mellifera	*CYP9Q3*	32.10
Apis cerana	*CYP9Q3*	3.11
Apis dorsata	*CYP9Q3*	20.74
Apis florea	*CYPQ2*	77.15
Apis florea	*CYP9Q19*	33.89
Eufriesea mexicana	*CYP9Q8*	35.63
Eufriesea mexicana	*CYP9Q7*	25.15
Melipona quadrifasciata	*CYP9Q10*	74.46
Tetragonula carbonaria	*CYP9Q17*	36.32
Tetragonula carbonaria	*CYP9Q16*	77.71
Habropoda laboriosa	*CYP9Q9*	56.04
Xylocopa violacea	*CYP9Q18*	109.76
Melitta haemorrhoidalis	*CYP9FU3*	28.34
Colletes cunicularius	*CYP9FZ2*	19.98
Andrena haemorrhoa	*CYP9FT1*	n.d.
Lasioglossum xanthopus	*CYP9DL6*	31.38
Bombus impatiens	*CYP9Q6*	n.d.
Bombus impatiens	*CYP9Q5*	59.64
Bombus impatiens	*CYP9Q4*	28.67
Macropis fulvipes	*CYP9FU2*	88.31
Dufourea novaeangliae	*CYP9DL4*	27.21
Eucera nigrescens	*CYP9Q15*	37.27
Nomada lathburiana	*CYP9Q14*	79.08
Nomia melanderi	*CYP9DL5*	123.40
Andrena vaga	*CYP9FT2*	8.68

Table S7. Steady-state kinetics data for BOMFC metabolism resulting in 7-hydroxy-4-(trifluoromethyl)coumarin (HC) by recombinantly expressed bee P450s of five subfamilies co-incubated with increasing concentrations of flupyradifurone (FPF). The inhibition type is based on kinetic characteristics of reversible inhibition models according to Fowler and Zhang (*The AAPS Journal* **10** (2008) 410-424).

P450 ± [FPF] (Inhibition type)	K_m [µM]	95% CI	V_{max} pmol HC / min * mg protein	95% CI	Adjusted R^2
CYP9Q18 (non-competitive)					
w/o	15.2	11.6 to 19.8	171	158 to 186	0.94
10 µM	14.2	10.9 to 18.6	155	142 to 168	0.93
30 µM	15.2	11.7 to 19.7	134	123 to 145	0.94
100 µM	16.9	12.9 to 22.3	92	84.5 to 100	0.93
CYP9FU3 (non-competitive)					
w/o	8.16	6.36 to 10.4	189	176 to 202	0.94
10 µM	8.71	6.78 to 11.2	190	177 to 203	0.94
30 µM	9.93	7.71 to 12.8	183	170 to 196	0.94
100 µM	12.2	9.27 to 16.1	155	143 to 168	0.93
CYP9FZ2 (non-competitive)					
w/o	14.7	11.1 to 19.4	69.0	63.3 to 75.1	0.93
10 µM	15.8	11.9 to 21.0	63.0	57.6 to 68.8	0.93
30 µM	17.3	13.2 to 22.6	56.0	51.4 to 61.1	0.93
100 µM	20.5	15.6 to 27.1	38.5	35.1 to 42.2	0.93
CYP9DL4 (non-competitive)					
w/o	11.0	9.27 to 13.0	78.0	74.4 to 81.8	0.97
10 µM	12.9	10.7 to 15.5	82.5	77.6 to 87.8	0.97
30 µM	12.7	10.4 to 15.4	74.9	70.7 to 79.3	0.96
100 µM	18.3	15.1 to 22.3	65.0	61.5 to 68.8	0.97
CYP9FT2 (non-competitive)					
w/o	7.43	5.65 to 9.74	50.6	46.9 to 54.4	0.92
10 µM	10.1	8.02 to 12.8	49.9	46.7 to 53.3	0.95
30 µM	10.4	8.18 to 13.2	46.5	43.4 to 49.8	0.95
100 µM	10.8	8.26 to 14.1	42.2	39.0 to 45.6	0.93

Table S8. Incubation assays (2h) with recombinantly expressed CYP9Q orthologs and subsequent UPLC-MS/MS analysis resulting in a significant disparity between TCP-depletion and TCP-OH formation, suggesting additional metabolites are formed that are not detected in our analysis (values in pmol per mg protein) (unpaired t-test $p < 0.05$).

**Enzyme**	**P value**	**Mean of TCP Depletion**	**Mean of TCP-OH**
CYP9FU2 M. fulvipes	0.000039	5191	450.1
CYP9Q3 A. mellifera	0.000088	9092	6728
CYP9FU3 M. haemorrhoidalis	0.000242	5060	1302
CYP9Q15 E. nigrescens	0.001659	3177	441.5
CYP9Q14 N. lathburiana	0.003516	2121	255.2
AdCYP9Q3 A. dorsata	0.004755	3908	1658
CYP9Q18 X. violacea	0.005428	8323	2285
CYP9FT1 A. vaga	0.007835	1617	99.42
CYP9Q16 T. carbonaria	0.008379	1309	346.0
CYP9FZ2 C. cunicularius	0.010265	7683	7110
CYP9DL4 D. novaeangliae	0.012953	2432	1734
AfCYP9Q2 A. florea	0.015532	1819	318.2
CYP9Q8 E. mexicana	0.020614	6012	4615
CYP9Q17 T. carbonaria	0.024269	1168	51.48
CYP9DL5 N. melanderi	0.043334	3204	2498
CYP9Q10 M. quadrifasciata	0.044054	2745	1549

Table S9. Ion transitions and linear range of insecticides and their metabolites quantified by UPLC-MS/MS analysis.

Compound	Ion Transition	Linearity [ng / mL]
TCP	253 > 187	0.2 - 100
TCP-OH	269 > 202	0.1 - 100
IMD	256 > 175	0.1 - 200
IMD-OH	272 > 191	0.5 - 200
FPF	289 > 126	0.1 - 100
FPF-AF	164 > 146	0.3 - 50
FPF-DFEA	207 > 126	0.5 - 100
FPF-AA	265 > 126	0.2 - 200
FPF-OH	305 > 126	0.1 - 100
